# Efficacy of bimatoprost 0.03% in reducing intraocular pressure in patients with 360° synechial angle-closure glaucoma: A preliminary study

**DOI:** 10.4103/0301-4738.73708

**Published:** 2011

**Authors:** Prateep Vyas, Uday Naik, Jayasheel B Gangaiah

**Affiliations:** Department of Ophthalmology, Choithram Netralaya, Dhar Road, Indore - 01, India; 1 Manipal AcuNova Limited, Mobius Towers, SJR - I Park, EPIP Zone, Whitefield, Bangalore - 560 066, India

**Keywords:** Bimatoprost, chronic angle-closure glaucoma, intraocular pressure, peripheral anterior synechiae, peripheral iridotomy

## Abstract

**Context:**

Peripheral anterior synechiae (PAS; synechiae anterior to functional trabecular meshwork) formation in primary angle-closure glaucoma (PACG) hampers access to uveoscleral outflow. Thus, the role of bimatoprost in such patients with 360° synechiae was evaluated.

**Aims:**

To assess efficacy and safety profile of bimatoprost 0.03% in lowering intraocular pressure (IOP) in 360° synechial angle-closure glaucoma patients.

**Settings and Design:**

This was a prospective, non-randomized, non-comparative, selective analysis, single-center pilot study.

**Materials and Methods:**

A total of 23 eyes of 20 Indian chronic angle-closure glaucoma (CACG) patients with IOP greater than 21 mmHg, 360° PAS and no visual potential in the study eye underwent detailed eye examination. Baseline IOP was measured and YAG peripheral iridotomy was performed for complete angle-closure reconfirmation. Bimatoprost 0.03% was administered for 8 weeks as once-daily evening dose. IOP reduction within treatment group was determined with “paired t-test”.

**Results:**

The mean reduction in IOP from baseline to 8 weeks of bimatoprost therapy was 15.3 ± 9.5 mmHg (*P* < 0.001). The most commonly observed adverse event was conjunctival hyperemia (35%). Bimatoprost was well tolerated in the study.

**Conclusions:**

In this study, exclusively involving patients with 360° synechial angle-closure glaucoma and no visual potential, bimatoprost 0.03% treatment demonstrated a statistically significant IOP reduction. Hence, it can be inferred that bimatoprost 0.03% is an efficacious treatment modality in this subgroup of patients for reducing IOP.

Glaucoma, the second principal cause of worldwide blindness, affects 66.8 million people and results in 6.7 million bilaterally blinded people, thus contributing to 15% of global blindness.[1] As per the current statistical estimates, there are about 12 million glaucoma patients in India, and approximately 12.8% of the country’s blindness can be ascribed to it.[2]

Primary angle-closure glaucoma (PACG) is a major form of glaucoma in Asian countries,[3,4] with primary chronic angle-closure glaucoma (CACG) being highly prevalent in Indian eyes.[4] According to an Indian hospital-based data, PACG accounts for nearly 50% of the cases in India.[2]

Population-based epidemiological studies in India are limited and include three landmark studies: the Vellore eye survey,[5] the Andhra Pradesh eye disease survey[6] and the Aravind comprehensive eye survey,[7] which have reported a PACG prevalence of 4.32, 0.71 and 0.5%, respectively.

Though laser peripheral iridotomy (PI) relieves the pupillary block component in CACG, adjuvant anti-glaucoma medications are required to control the chronically raised intraocular pressure (IOP) caused by aqueous outflow obstruction secondary to synechial angle closure and trabecular meshwork damage.[8] Routinely used medications for the management of primary CACG include beta-blockers and pilocarpine.

Recent compelling evidence has showcased the well-sustained and good IOP lowering effects of prostaglandin analogues in CACG patients.[8–11] Bimatoprost, a synthetic analogue of prostamide F2α, is a potent ocular hypotensive and its efficacy in ocular hypertension (OHT), primary open-angle glaucoma (POAG),[9,12–16] PACG,[8,9,17] CACG,[18] exfoliative glaucoma[19] and normal tension glaucoma[20] has been well documented. Latanoprost, another synthetic prostaglandin analogue, has also shown efficacy in CACG.[21] However, peripheral anterior synechiae (PAS) formation in primary CACG patients may hamper access to the uveoscleral outflow and hence the role of bimatoprost in CACG with 360° PAS needs evaluation. To our knowledge, no preliminary study has evaluated the efficacy of bimatoprost 0.03% exclusively in CACG patients with 360° PAS and no visual potential. Hence, in this study, we contemplated to assess the efficacy of bimatoprost 0.03% in reducing IOP in CACG patients with no visible ciliary body face, no visual perception and abnormally high IOP.

## Materials and Methods

This prospective, non-randomized, non-comparative, selective analysis, single-center pilot study was performed to evaluate the IOP reducing efficacy of bimatoprost 0.03% in patients having primary CACG with 360° PAS, over a stipulated period of 8 weeks.

CACG was defined as primary angle closure associated with glaucomatous optic neuropathy, 360° PAS on gonioscopy and a chronically elevated IOP. The angular width of the anterior chamber angle recess was graded for all the four quadrants using Shaffer’s classification with dynamic gonioscopy. Eyes having 360° synechial angle closure without opening of any part of the angle were included for further study. For all the patient eyes enrolled in the study, iris was in contact with peripheral cornea, obscuring view of the anterior trabeculae as observed during the indentation gonioscopy procedure.

Twenty patients, aged 18 years and above, with established unilateral or bilateral CACG confirmed by indentation gonioscopy following neodymium (Nd) yttrium aluminium garnet (YAG) PI and with no visual potential in the study eye, were included consecutively. No visual potential comprised those patients having no hand movements and visual acuity ranging from inaccurate projection of rays (PR) to no perception of light (PL). For inclusion, patients also had to have a patent iridotomy done at least 4 weeks prior and a baseline IOP of >21 mmHg. Patients with secondary angle closure glaucoma were not included in the study.

Exclusion criteria comprised any prior intraocular surgery, any corneal abnormality preventing reliable applanation tonometry, evidence of visual potential, ocular infection or inflammation within 3 months of the screening visit, ocular therapy with a steroid or nonsteroidal anti-inflammatory drug within 1 month of the screening visit and evidence of open angle following YAG PI. Patients having uncontrolled systemic diseases, study drug sensitivity or medical conditions such as cardiac failure, sinus bradycardia, second and third degree atrioventricular block, bronchial asthma and chronic obstructive pulmonary disease were also excluded. All eyes used were virgin eyes, which were previously not on any systemic (carbonic anhydrase inhibitors) or topical anti glaucoma medication that could have a potential effect on measured IOP at the time of enrolment.

The preliminary evaluation during the screening visit consisted of medical and ocular history recording, vital parameters assessment, visual acuity assessment, slit-lamp examination, indentation gonioscopy with Sussman’s goniolens for differentiation between appositional and synechial closure, ophthalmoscopy and IOP measurement and recording by Goldmann applanation tonometer.

All eligible patients underwent Nd YAG laser PI. Post-iridotomy, IOP was measured after an hour and a course of steroid eye drops three times a day was prescribed for 5 days. The IOP recorded after three weeks of PI was considered as baseline IOP. At the baseline visit, re-confirmation of complete angle closure and patency of YAG PI was undertaken by indentation gonioscopy and slit-lamp examination, respectively. Visual acuity, ophthalmoscopy and rechecking for eligibility were also performed at the baseline visit. After obtaining written consent, all enrolled patients were given a vial of bimatoprost 0.03% with written and verbal instructions for accurate administration (one drop of study drug at 8:00 PM in the study eye) for a period of 8 weeks.

Post-treatment study visits were scheduled at day 1 and weeks 1, 4 and 8 of therapy. At each study visit, detailed examination for best corrected visual acuity, slit-lamp biomicroscopy, assessment of YAG PI opening patency, indentation gonioscopy examination, vital parameter assessment and IOP recording in triplicate at 8:00 AM, 10:00 AM and 4:00 PM were performed.

One or both the eyes of each subject were analyzed. The change in daily IOP from baseline to 8 weeks was analyzed; the daily IOP was calculated as the mean of the 8:00 AM, 10:00 AM and 4:00 PM IOP values. Reduction of IOP within treatment group was determined with “paired *t*-test.”

The study was conducted after getting approval from the institutional ethics committee. The procedures followed in the study were in accordance with the ethical standards and with the Helsinki Declaration of 1975, revised in 2000.

## Results

A total of 23 eyes of 20 patients with 360° synechial CACG were included for the study. All patients were of Indian ethnicity and all of them completed the study. The mean age of the patients participating in the study ranged from 40 to 70 years with a mean of 56.15 ± 8.24 years. There were 13 (65%) females and 7 (35%) males in the study. Seventeen (85%) patients had unilateral CACG and 3 (15%) subjects had bilateral affection of eyes. For the visual acuity, 17 (74%) had inaccurate PR while 6 (26%) patients had no PL.

YAG laser PI was done in 20 eyes (87%) of 17 patients and in the remaining three eyes (13%) of three patients, PI was not performed as they had 360° sphincter atrophy and dilated fixed pupils. The pre- and post-PI IOPs were 44.1 ± 4.4 and 43.2 ± 5.2 mmHg, respectively.

The pre-treatment mean baseline IOP was 43.2 ± 5.2 mmHg. After 8 weeks of treatment, mean IOP was significantly reduced with the post-treatment mean IOP at day 1, weeks 1, 4 and 8 being 27.4 ± 9.7, 29.7 ± 10.6, 29.0 ± 9.3 and 28.0 ± 9.9 mmHg, respectively [Table 1]. The overall mean fall in IOP following 8 weeks bimatoprost therapy was 15.3 ± 9.5 mmHg (*P* < 0.001). The mean percentage reduction in IOP from baseline to 8 weeks of bimatoprost therapy was 35.0%. Percentage reduction in IOP following therapy from baseline is summarized in Table 1. Two patients showed improvement in vision. Seventeen eyes showed more than 20% reduction in IOP, five eyes showed <20% reduction in IOP and one patient did not show any response to the medication.

**Table 1:** Mean post-treatment IOP and percentage reduction in IOP from baseline to 8 weeks in the study group

Visits		Time points			Mean IOP* at each visit and average percentage reduction in IOP
		8.00 AM	10.00 AM	4.00 PM	
Visit 1	Mean IOP	27.2 ± 8.5	27.1± 11.1	28.0 ± 9.9	27.4 ± 9.7
Day 1	% Reduction in IOP	37.3	37.8	35.6	36.9
Visit 2	Mean IOP	29.0 ± 9.9	29.7 ± 11.5	30.5 ± 10.6	29.7 ± 10.6
Week 1	% Reduction in IOP	33.0	31.6	29.9	31.5
Visit 3	Mean IOP	29.7 ± 10.3	28.9 ± 9.7	28.3 ± 8.0	29.0 ± 9.3
Week 4	% Reduction in IOP	30.6	32.6	34.5	32.6
Visit 4	Mean IOP	27.8 ± 10.4	27.7 ± 9.7	28.6 ± 10.0	28.0 ± 9.9
Week 8	% Reduction in IOP	35.5	35.9	33.6	35.0

* Intraocular pressure measured in mmHg; mean of three IOP readings at each time-point was recorded for each patient

The most commonly reported adverse event was conjunctival hyperemia seen in 7 out of 20 patients (35%). Common adverse effects observed during 8 weeks of bimatoprost therapy were punctate epetheliopathy seen in 1 (5%) patient and foreign body sensation seen in 2 (10%) patients. Vital parameters were unaffected with no systemic side effects observed.

## Discussion

As there is a higher POAG preponderance in Caucasian eyes, there is comparatively a paucity of global literature available on CACG. However, Indian epidemiological data have emphasized on the potential vision-threatening implications of having a higher prevalence of PACG in the Indian population.[2] Primary CACG is a potentially vision-impairing condition and is complicated by its subtle and asymptomatic clinical presentation. Prompt and early institution of interventions such as surgery and IOP lowering medications are crucial to prevent additional damage and to salvage the patient’s vision. Prostaglandin analogues are currently the most efficacious and safe drugs for lowering IOP in glaucoma. A considerable number of reviews and trials have revealed the higher IOP reducing potency of the newer prostaglandin analogue, bimatoprost, as compared to latanoprost.[22,23] As such, the purpose of this study was to evaluate the efficacy of 8 weeks of bimatoprost therapy in primary CACG patients with 360° PAS and no visual potential. The trial included exclusively the subjects having no visual potential as the role of bimatoprost on vision has not been clearly elucidated and, moreover, bimatoprost monotherapy would not suffice for lowering very high IOP and hence its consequent effect on sight of patients having useful vision is uncertain.

This study included 20 primary CACG patients with 360° synechial angle closure inadequately controlled with YAG PI (pre-PI IOP of 44.1 + 4.4 mmHg to post-PI IOP of 43.22 + 5.2 mmHg; *P* = 0.604). The mean age of presentation was 56.15 ± 8.24 years (range 40–70 years), with females constituting about 65%. Studies performed in Asian[9] and Indian[2,4] primary CACG populations have shown a mean age at presentation of CACG in the sixth or seventh decade, with a marginally higher preponderance in females. From these reports it can be corroborated that Indians, especially women, are at a higher risk of PACG development and consequent early CACG progression.

It has been postulated that in comparison to other ethnicities, Asian irides are thicker and stickier and therefore tend to form PAS more easily.[24] As the role of bimatoprost in CACG patients is not clearly discerned, it becomes imperative to elucidate its precise mechanism of action in this subset of patients. Apparently, bimatoprost is perceived to exert its effect by enhancing uveoscleral outflow,[25–28] by remodeling of extracellular matrix by induction of matrix metalloproteinase activity[29] and by ciliary muscle relaxation. The degree and extent of PAS could thus impede the aqueous outflow into the uveoscleral pathway and in turn may affect the IOP lowering efficacy of prostaglandin analogues.

Our study results demonstrated a statistically significant (*P* < 0.001) IOP lowering efficacy of bimatoprost 0.03% in CACG patients, with the mean reduction in IOP from baseline to 8 weeks of therapy being 15.3 ± 9.5 mmHg (43.2 ± 5.2 to 28.0 ± 9.9 mmHg). In similar clinical trials conducted by Chen *et al*.[8] (8 weeks), the percentage fall in IOP from baseline to 8 weeks of bimatoprost therapy was 25.8%. Thus, our study result is consistent with earlier observations of fall in IOP of about 25–35% in patients with POAG, OHT[9,12,15,17] and primary CACG[8,9] treated with bimatoprost. Further, the results project the uniform and persistent diurnal IOP reducing capacity of bimatoprost in CACG patients.

These reports and our study result help assuage speculations concerning the efficacy of bimatoprost in CACG patients with 360° angle closure.

Bimatoprost was recognized to be safe and well tolerated in this trial. Conjunctival hyperemia was the most commonly reported side effect observed in 7 out of 20 patients (35%), followed by foreign body sensation observed in 2 out of 20 patients (10%) and punctate epitheliopathy observed in 1 out of 20 patients (5%). The occurrence of conjunctival hyperemia was observed to be in accordance with current existing literature[8,9] and was of a mild and transient nature, not associated with intraocular inflammation or other sequel. This may also be the reason for no discontinuations in the study. Other side effects such as change in eyelash color and size, change in the iris pigmentation and change in the periocular pigmentation were not observed, probably owing to the short duration of the study period. Further, bimatoprost was found to be systemically safe with vital parameters remaining unaffected.

We observed unanticipated improvement in vision in two patients (one of them improved to 20/200 and the other to 20/50 in both eyes). This could probably be ascribed to augmentation of ocular blood flow by bimatoprost in patients with CACG, which may occur due to acute reduction of IOP.[30] However, larger randomized trials are needed for establishing a conclusive relationship between increased ocular blood flow and improvement of sight following bimatoprost therapy in CACG patients. One eye (4%) did not show reduction in IOP; this could be a case of nonresponder to PG analogue. This finding is similar to a study which showed about 4% nonresponders to bimatoprost.[19]

Although the results of the study demonstrated the efficacy of bimatoprost in IOP reduction, this trial has a few limitations which include short study duration and small sample size. Hence, although the IOP reducing efficacy of bimatoprost is established in this subset of patients on the basis of this preliminary study, larger randomized controlled trials are warranted for pragmatic decision making.
